# Loss of wobble uridine modification in tRNA anticodons interferes with TOR pathway signaling

**DOI:** 10.15698/mic2014.12.179

**Published:** 2014-11-29

**Authors:** Viktor Scheidt, André Jüdes, Christian Bär, Roland Klassen, Raffael Schaffrath

**Affiliations:** 1 Institut für Biologie, Abteilung Mikrobiologie, Universität Kassel, D-34132 Kassel, Germany.; 2 Present address: Molecular Oncology Program, Spanish National Cancer Centre (CNIO), Melchor Fernandez Almagro 3, Madrid, Spain.

**Keywords:** Saccharomyces cerevisiae, TOR signaling, rapamycin, Gln3, NCR, Sit4, Elongator complex, tRNA anticodon modification, tRNase zymocin

## Abstract

Previous work in yeast has suggested that modification of tRNAs, in particular uridine bases in the anticodon wobble position (U34), is linked to TOR (target of rapamycin) signaling. Hence, U34 modification mutants were found to be hypersensitive to TOR inhibition by rapamycin. To study whether this involves inappropriate TOR signaling, we examined interaction between mutations in TOR pathway genes (*tip41*∆,* sap190*∆,* ppm1*∆,* rrd1*∆) and U34 modification defects (*elp3*∆, *kti*12∆,* urm1*∆,* ncs2*∆) and found the rapamycin hypersensitivity in the latter is epistatic to drug resistance of the former. Epistasis, however, is abolished in tandem with a* gln3*∆ deletion, which inactivates transcription factor Gln3 required for TOR-sensitive activation of NCR (nitrogen catabolite repression) genes. In line with nuclear import of Gln3 being under control of TOR and dephosphorylation by the Sit4 phosphatase, we identify novel TOR-sensitive sit4 mutations that confer rapamycin resistance and importantly, mislocalise Gln3 when TOR is inhibited. This is similar to *gln3*∆ cells, which abolish the rapamycin hypersensitivity of U34 modification mutants, and suggests TOR deregulation due to tRNA undermodification operates through Gln3. In line with this, loss of U34 modifications (*elp3*∆, *urm1*∆) enhances nuclear import of and NCR gene activation (*MEP2*, *GAP1*) by Gln3 when TOR activity is low. Strikingly, this stimulatory effect onto Gln3 is suppressed by overexpression of tRNAs that usually carry the U34 modifications. Collectively, our data suggest that proper TOR signaling requires intact tRNA modifications and that loss of U34 modifications impinges on the TOR-sensitive NCR branch via Gln3 misregulation.

## INTRODUCTION

While cell growth and proliferation are typically characterized by active *de novo* protein synthesis, cell quiescence goes along with translational downregulation. So, translational activity and metabolic cycling need to be tightly regulated in response to growth signals 1. In eukaryotes including budding yeast, this is coordinated by the nutrient-sensitive TOR (target of rapamycin) kinase pathway, which among others promotes the biogenesis of components essential for translation, i.e. ribosomal proteins, rRNAs and tRNAs 1,2. During maturation, tRNAs undergo many posttranscriptional modifications which appear to occupy roles in TOR-dependent processes 3,4,5. In line with this notion are TOR-indicative defects in filamentous growth and metabolic cycling as well as GAAC (general amino acid control) response signatures typical of tRNA modification mutants including those that fail to form 5-methoxycarbonylmethyl-2-thiouridine (mcm5s2U) onto anticodon wobble uridines (U34) 5,6,7. The mcm5s2U34 modification depends on two pathways one of which requires the Elongator complex (Elp1-Elp6) for mcm5 side chain formation while the second one (Uba4, Urm1, Ncs2/Ncs6) provides S-transfer for s2 thiolation 8,9,10. In support of a link between TOR signaling and tRNA modification, Elongator and thiolation mutants are hypersensitive to TOR inhibition by caffeine and rapamycin 10,11,12,13,14.

When TOR activity is low due to nitrogen-starvation (or rapamycin treatment), type 2A protein phosphatases (PP2A) including Sit4 are typically freed from TOR control and dephosphorylate TOR pathway targets including Gln3 1,2. Gln3 is required for transcription of NCR (nitrogen catabolite repression) genes and its release from TOR phosphoinhibition by Sit4 dephosphorylation leads to its nuclear import and NCR gene activation 2,15. Consistent with its TOR-sensitive role, loss of Gln3 causes rapamycin resistance. Gln3 activation also involves genes that encode regulators of PP2A including Sit4 (*TAP42*, *TIP41*, *SAP190*, *PPM1/2*, *RRD1/2*) and that, when mutated, protect against rapamycin 2,15,16,17,18. Among these, the *RRD1/2 *products are PPIases (peptidyl-prolyl *cis*/*trans*-isomerases) which bind and activate PP2A enzymes through conformational changes thought to confer substrate specificity 19,20,21. Consistent with such activator role for Sit4, *rrd1/2*∆ mutants accumulate Gln3 in its phosphorylated form 22. Yet, *sit4*∆ cells are hypersensitive to rapamycin and this trait is unaltered in tandem with *rrd1/2*∆ null-alleles, which alone cause drug resistance 17,22,23. This implies that the hypersensitivity of *sit4*∆ cells is independent of Rrd1/2 function. A TOR-independent role for Sit4 is indeed known, and was shown to confer growth inhibition by zymocin 17,24, a lethal tRNase toxin which kills cells by cleaving mcm5s2U34 modified anticodons (see above). Hence, tRNA modification defects typical of Elongator and *sit4*∆ mutants cause zymocin resistance 8,12,24,25,26. *sit4*∆ mutants accumulate hyperphosphorylated Elongator forms demonstrating a link between Sit4 dephosphorylation and Elongator’s tRNA modification function 27,28. This Sit4 role is independent of Rrd1/2 but requires the Sit4 partner proteins Sap185 and Sap190 17,23,29, which is why a *sap185*∆*190*∆ double mutant copies defects typical of *sit4*∆ and Elongator mutants (i.e. Elongator hyperphosphorylation, loss of tRNA modification, zymocin resistance and rapamycin hypersensitivity) 25,26,27,28.

Here, we show that the rapamycin hypersensitivity of Elongator and U34 thiolation mutations (*elp3*∆,* urm1*∆), which are epistatic to TOR signaling mutations (*tip41*∆, *sap190*∆, *ppm1*∆, *rrd1*∆), is suppressed by overexpression of tRNAs known to undergo U34 modification and entirely abolished by a *GLN3* deletion. This implies improper tRNA functioning due to loss of anticodon modifications interferes with the TOR signaling pathway through Gln3. Consistently, we can correlate mislocalisation of and upregulated NCR gene (*MEP2*, *GAP1*) activation by Gln3 with tRNA anticodon (U34) modification defects. This strongly suggests tRNA modifications are required for proper signaling into TOR-sensitive activation of NCR genes and regulation of Gln3.

## RESULTS AND DISCUSSION

**Figure 1:**
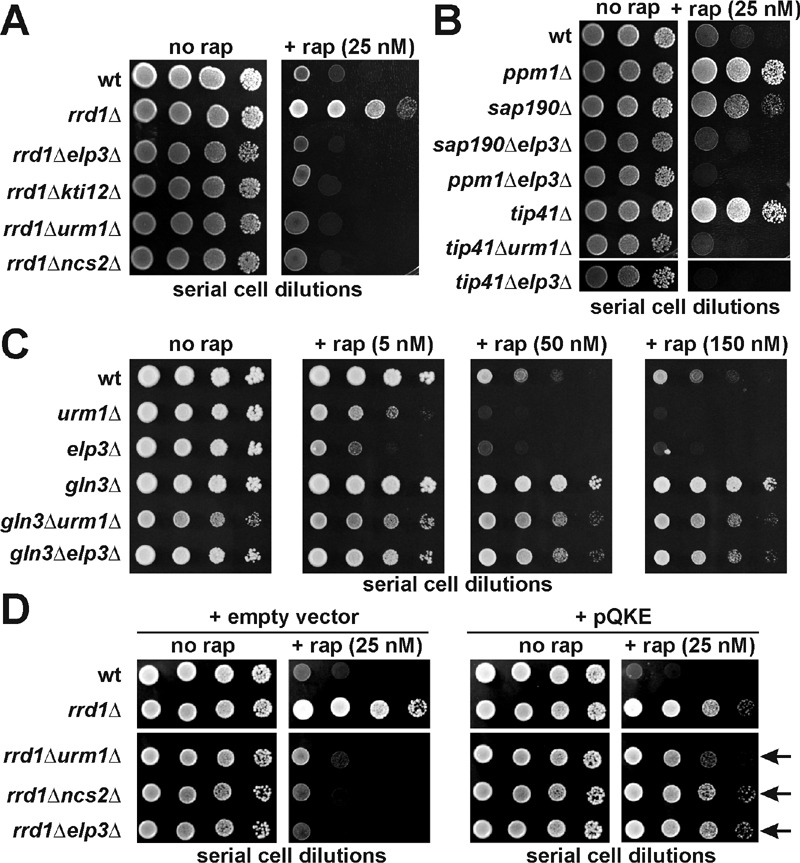
FIGURE 1: Genetic interaction between mutations in TOR signaling and tRNA modification pathways. **(A) **Rapamycin hypersensitivity due to loss of tRNA modification in Elongator and U34 thiolation mutants is epistatic over TOR signaling mutant *rrd1*∆. **(B) **Other mutations in TOR pathway signaling genes cannot alter the rapamycin sensitive phenotype of Elongator or U34 thiolation mutants. **(C) **Hypersensitivity of U34 modification mutants to TOR inhibition by rapamycin requires Gln3 function. **(D) **Overexpression of tRNA species (tRNA^Gln^, tRNA^Lys^ and tRNA^Glu^) known to undergo U34 anticodon modification suppresses the rapamycin sensitivity of *rrd1*∆ cells carrying tRNA modification defects and reinstates drug tolerance. Shown are drug responses with vector controls (+ empty vector: left panels) or in response to tRNA overexpression from multicopy plasmid (+ pQKE: right panels) carrying tRNA^Gln^ (Q) tRNA^Lys ^(K) and tRNA^Glu^ (E) genes. Phenotypic suppression is indicated by arrows. In (A-D), ten-fold serial dilutions of yeast tester strains with genetic backgrounds as indicated were spotted onto YPD media containing no drug (no rap) or various doses (5, 20, 25, 50 and 150 nM) of rapamycin (+ rap) and grown for 3-5 days at 30°C. Lack of growth indicates rapamycin sensitive or hypersensitive responses while growth in the presence of the TOR inhibitor drug equals rapamycin resistance.

Previously, it was shown that inactivation of either Sit4 (*sit4*∆) or all four Sap proteins (*sap4*∆*155*∆*185*∆*190*∆) required for Sit4 function causes rapamycin hypersensitivity and that this trait is not altered in combination with an *rrd1*∆ null-allele 17,23. Intriguingly, reintroduction of *SAP190* into *rrd1*∆*sap*∆∆∆∆ cells reestablishes the drug resistance typical of *rrd1*∆ cells alone suggesting the trait depends on Sit4 and Sap190 17. This is similar to Sit4 dependent Elongator dephosphorylation, which requires Sap185 and Sap190 and promotes Elongator’s tRNA modification function 25,27,28. Elongator dephosphorylation can be suppressed by high dosage of *KTI12*, a gene coding for a potential regulator of Elongator and Sit4 12,27. Strikingly, multicopy *KTI12* gene dosage was also found to confer rapamycin sensitivity in an *rrd1*∆ background (Supplemental Figure 1). With Kti12 being intimately linked to Elongator 8,12,30,31, we observed that both Elongator and *KTI12* gene deletions (*elp3*∆, *kti12*∆) phenocopied each other and caused *rrd1*∆ cells to become rapamycin sensitive (Figure 1A). Besides Elongator function, formation of the mcm5s2U34 modification also requires components of the U34 thiolation pathway (i.e. Urm1, Uba4 and Ncs2/Ncs6) 8,9,10. When we combined sulfur transfer defects (*urm1*∆, *ncs2*∆) with an *rrd1*∆ allele, the resulting drug sensitivity basically copied the above epistasis seen with Elongator and *kti12 *mutations (Figure 1A). Similarly, on studying growth inhibition by caffeine, a TOR inhibitor drug distinct from rapamycin 32, we again found epistasis between U34 modification defects and an *rrd1*∆ null-allele confirming that the genetic interactions seen were specific to the TOR pathway (Supplemental Figure 2).

Next, we included TOR signaling mutants with defects in the Gln3 pathway (*sap190*Δ, *tip41*Δ or *ppm1*Δ) 15,18 into our assays. We found that on their own, they copied the rapamycin resistance of *rrd1*∆ cells, but in tandem with an *elp3*Δ mutation, they also failed to counter the drug sensitivity typical of Elongator mutants (Figure 1B). Consistent with our observation that the drug sensitivity of thiolation defects was independent of *rrd1*Δ (Figure 1A), we observed that the rapamycin hypersensitivity of an *urm1*Δ mutant was not altered in tandem with a *tip41*Δ null-allele, which alone is a potent rapamycin suppressor (Figure 1B). Taken together, our data indicate that TOR-related phenotypes (rapamycin and caffeine hypersensitivity), which result from loss of U34 modifications in Elongator and thiolation mutants, are not modulated by Gln3 pathway gene mutations that cause protection against TOR inhibitor drugs.

Gln3 is a TOR-sensitive transcription factor necessary for NCR gene activation 33. We found that the rapamycin resistance of a *gln3*Δ mutant was hardly altered in tandem with either *elp3*Δ or *urm1*Δ null-alleles (Figure 1C). So in striking contrast to the above TOR signaling mutants, the drug phenotype of *gln3*Δ cells is insensitive to tRNA modification defects indicating the rapamycin hypersensitivity of *elp3*Δ and *urm1*Δ mutants requires Gln3 function. Activity of Gln3 is largely regulated at the level of nucleo-cytoplasmic shuttling and cytosolic localization involves phosphoinhibition of Gln3 by TOR and interaction with Ure2 for cytosolic retention 34. Consistently, inactivation of Ure2 in *ure2*Δ cells leads to nuclear import of Gln3, NCR gene activation and rapamycin hypersensitivity 34. Having found that Gln3 is critical for the rapamycin hypersensitivity of U34 modification mutants, we asked if this trait may involve *URE2* function. On comparing rapamycin phenotypes between *ure2*Δ and *urm1*Δ single as well as *ure2*Δ*urm1*Δ double mutants, we observed a hypersensitive response of the latter to the TOR inhibitor drug (Supplemental Figure 3). Such phenotype suggests that in combination, defects in Ure2 and U34 modification are additive and result in enhanced sensitivity to TOR inhibition by rapamycin.

Studies in yeast have shown that U34 tRNA modification defects and associated phenotypes are suppressible by overexpressing tRNAs that would normally undergo the U34 anticodon modifications. Therefore, we repeated the above assays in the absence (empty vector) and presence of a multicopy plasmid (pQKE) that allows for overexpression of tRNAs (tRNA^Gln^ [Q], tRNA^Lys^ [K] and tRNA^Glu^ [E]) known to be mcm5s2U34 modified in wild-type cells (see above). As illustrated in Figure 1D, higher-than-normal levels of these tRNA species had efficient suppressor function and reconferred a rapamycin tolerant trait to *rrd1*∆*elp3*∆, *rrd1*∆*urm1*∆ and *rrd1*∆*ncs2*∆ double mutants that almost compares to the drug tolerance of an *rrd1*∆ single mutant alone. Similarly, we found that the caffeine sensitive phenotype typical of the *rrd1*∆*elp3*∆ double mutant was suppressed by tRNA overexpression (Supplemental Figure 4) to confer a drug tolerant trait resembling the *rrd1*∆ mutant alone. Taken together, these suppression data strongly suggest that the effect U34 modification defects obviously have on TOR pathway mutants including *rrd1*∆ cells can be ascribed to loss of Elongator’s tRNA modification function (*elp3*∆) and deficient thiolation (*urm1*∆, *ncs2*∆). So, proper tRNA anticodon (U34) modification and translation-related tRNA functions that are associated with it are apparently required for intact TOR pathway signaling.

**Figure 2:**
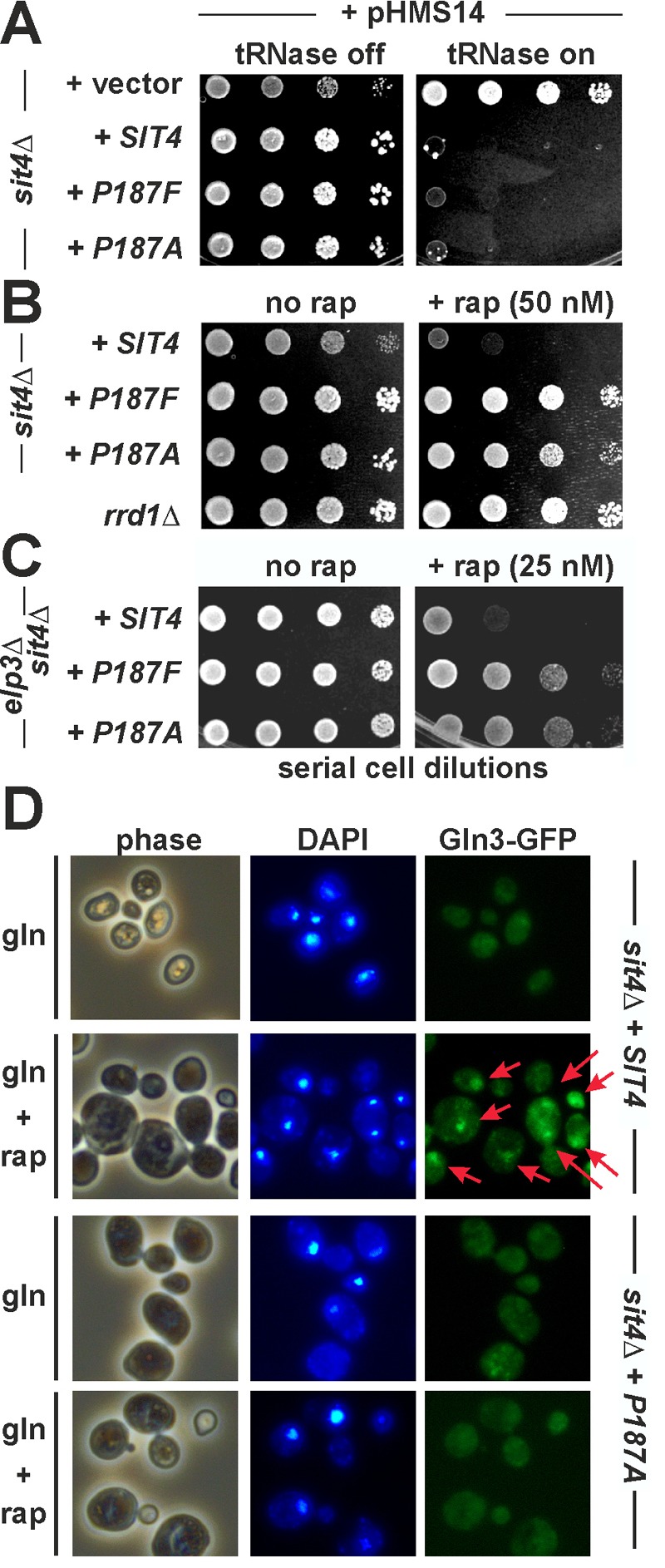
FIGURE 2:Substitutions of Sit4 proline residue 187 identify novel *sit4* mutants that separate TOR-dependent phosphatase functions from TOR-insensitive ones. **(A)** TOR-independent zymocin γ-toxin tRNase assay. The indicated *sit4*∆ backgrounds carrying empty vector, wild-type *SIT4* and the P187F/A substitution alleles were transformed with the *GAL1*::γ-toxin expression vector (pHMS14) 12 and spotted onto glucose repressing (tRNase off) or galactose inducing (tRNase on) media. Growth was for 3 days at 30°C. Resistance or sensitivity towards tRNase toxicity is distinguished by growth or lack of growth, respectively. **(B)** TOR-sensitive rapamycin phenotype. Ten-fold serial cell dilutions of a TOR signaling mutant (*rrd1*∆) and *sit4*∆ cells with genetic backgrounds as indicated in (A) were spotted onto medium containing rapamycin (+ rap, 50 nM) or no drug (no rap). Lack of growth indicates drug sensitivity, growth equals rapamycin resistance. **(C)** The novel *sit4* separation of function mutations suppress the sensitivity of Elongator mutants to rapamycin. A *sit4*∆*elp3*∆ double mutant with genetic backgrounds as indicated in (A) was grown on plates with no drug (no rap) or containing rapamycin (+ rap, 25 nM). **(D) **Gln3 mislocalises as a result of the P187 substitution and fails to be imported into the nucleus under conditions of TOR inhibition. Cells carrying pRS416-GFP-Gln3 were grown in minimal medium containing glutamine (gln) as the sole N-source with or without 10 nM rapamycin (rap) and images taken in phase contrast, DAPI- and GFP-fluorescence modes (phase, DAPI, Gln3-GFP). Arrows indicate GFP signals and foci that co-localise with DAPI-stained nuclei.

With Gln3 localisation involving dephosphorylation by Sit4, we next asked whether TOR-dependent roles of this multifunctional PP2A phosphatase could be distinguished from TOR-independent ones. Mammalian PP2A phosphatase activator (PTPA) and yeast Rrd1/2 are related PPIases with roles in phosphatase regulation 18,19,20,21,22,35. Isomerization of human PP2A by PTPA involves a proline residue (P190) 19 which in yeast Sit4 highly likely aligns to P187. To study the significance of P187, substitutions (P187A/F) were generated and analysed in zymocin and rapamycin assays indicative for TOR-independent and TOR-dependent Sit4 functions 12,17,18. As for the zymocin assays, a *SIT4 *wild-type allele, the substitutions (P187A/F) and empty vector were co-transformed into a *sit4*Δ reporter strain with pHMS14. pHMS14 allows for galactose-inducible expression of the tRNase γ-toxin subunit of zymocin, which in the presence of active Sit4 (and Elongator’s intact tRNA modification function) cleaves anticodons and becomes lethal 25,36,37. Upon galactose induction, *sit4*Δ cells (with empty vector) survived tRNase expression. The *SIT4* wild-type cells and both substitution (P187A/F) mutants, however, became killed (Figure 2A) indicating that P187 is dispensable for the role Sit4 plays in zymocin inhibition and Elongator’s tRNA modification function. In contrast, the rapamycin assays (Figure 2B) show that, similar to *rrd1*Δ cells, the *sit4 *mutants (P187A/F) are resistent against TOR inhibition by the drug. Based on these novel differential *sit4 *phenotypes, the substitutions (P187A/F) thus separate TOR-independent (zymocin action) from TOR-dependent (rapamycin inhibition) Sit4 functions. Remarkably, the novel *sit4 *mutations suppressed the rapamycin hypersensitivity of *elp3*Δ cells (Figure 2C). This is similar to the *gln3*Δ scenario (Figure 1C) and suggests that in the P187A/F mutants, non-functional Gln3 may be responsible for this *elp3*Δ suppressor effect. To address this issue in more detail, we found that in relation to *SIT4* wild-type cells, the P187A/F mutants indeed mislocalised a GFP-Gln3 reporter and failed to shuttle the transcription factor into the nucleus following TOR inhibition by rapamycin (Figure 2D). So, cytosolic accumulation of GFP-Gln3 under conditions of TOR inactivation is in good agreement with our data showing that the P187A/F mutants evoke rapamycin resistance (Figure 2B), a trait indicative for Gln3 inactivation and copied by loss of Gln3 function in the *gln3*Δ null-mutant (Figure 1C).

**Figure 3:**
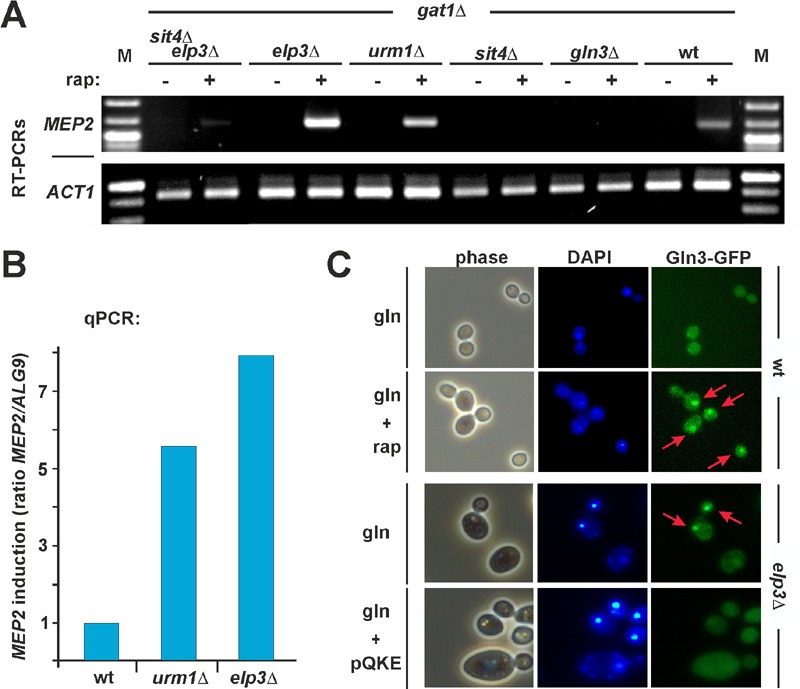
FIGURE 3: Loss of U34 anticodon modification causes nuclear Gln3 mislocalisation and enhances TOR-sensitive gene activation by Gln3. **(A, B)** RT-PCR (A) and qPCR (B) reveal that the TOR-sensitive *MEP2* gene transcription by Gln3 is enhanced in anticodon modification mutants that lack Elongator and U34 thiolation activities. Total RNA was isolated from the indicated strains cultivated with good nitrogen source (glutamine) supply in the absence (-) or presence (+) of 50 mM (rap) rapamycin. Following RT-PCR, the transcriptional induction of the *MEP2 *gene was analysed in comparison to actin (*ACT1*) transcription (A) and quantified by qPCR in relation to *ALG9* transcription (B) (mean values of triplicates). **(C) **Gln3 mislocalises to the nucleus in an *elp3*∆ Elongator mutant, a property suppressible by tRNA overexpression. Wild-type cells carrying pRS416-GFP-Gln3 were grown in minimal medium containing glutamine (gln) as the sole N-source with or without 10 nM rapamycin (rap). For localization studies with the Elongator mutant in response to tRNA overexpression, *elp3*∆ cells carrying pRS416-GFP-Gln3 together with empty vector control or multicopy tRNA plasmid (pQKE) were used and images taken in phase contrast, DAPI- and GFP-fluorescence modes (phase, DAPI, Gln3-GFP). Arrows indicate nuclear localization of GFP-tagged Gln3.

Therefore, we next asked whether the loss of tRNA modification in *elp3*Δ or *urm1*Δ mutants may interfere with TOR signaling through effects onto Gln3. Using RT-PCR and qPCR, we studied TOR-sensitive transcription of NCR genes (*GAP1, MEP2*) by Gln3 in response to alterations of tRNA anticodon modification. The strains were either wild-type or deleted for *ELP3 *or *URM1 *or they carried *sit4*Δ (and *sit4*Δ*elp3*Δ) or *gln3*Δ null-alleles in which phosphorylated Gln3 should remain cytosolic due to Sit4 defects (*sit4*Δ, *sit4*Δ*elp3*Δ) or no NCR gene activation should occur in the first place (*gln3*Δ). Also, all strains were *gat1*Δ to eliminate gene activation by a transcription factor redundant to Gln3. As Gln3-independent controls, we monitored actin *ACT1* (RT-PCR) and *ALG9* (qPCR) gene transcription 38. Under good nitrogen conditions (glutamine) and in the absence of rapamycin, we hardly detected any *MEP2 *and *GAP1 *activation by Gln3 in the tester strains (Figure 3A and Supplemental Figure 5A). This is consistent with phosphoinhibition by TOR and cytosolic localisation of Gln3 22,33,34. As expected, no *MEP2 *transcription was seen in *gln3*Δ, *sit4*Δ or *sit4*Δ*elp3*Δ mutants (Figure 3A). Consistent with Gln3 being released from TOR control by rapamycin, addition of the drug triggered basal gene activation by Gln3 (Figure 3A and Supplemental Figure 5A). Strikingly, however, *MEP2 *and* GAP1 *gene transcription was significantly upregulated in *elp3*Δ or *urm1*Δ cells (Figure 3A and Supplemental Figure 5A) and based on validation by qPCR 38, *MEP2* transcription increased roughly eight-fold (*elp3*Δ) and six-fold (*urm1*Δ) in relation to wild-type cells with proper tRNA modifications (Figure 3B). The finding that gene activation by Gln3 is significantly enhanced in the tRNA modification mutants is in line with our data showing that rapamycin hypersensitivity of *elp3*Δ or *urm1*Δ cells does require *GLN3 *function (Figure 1C). However, it is noteworthy that irrespective to TOR inhibition by rapamycin, even under conditions of TOR suppression by a poor nitrogen source (proline), constitutive Gln3-dependent transcription apparently differs between U34 modification mutants and wild-type cells (Supplemental Figure 5B).

As for conditions of good nitrogen (glutamine) supply, our RT-PCR data show that enhanced *MEP2* activation strictly depends on *SIT4 *function (Figure 3A), strongly suggesting that the transcription activation effects of U34 modification defects on Gln3 operate through the Sit4 phosphatase and require Gln3 dephosphorylation for nuclear localisation. Consistent with this notion, we observed that in the absence of TOR inhibition by rapamycin and in drastic contrast to wild-type cells, GFP-tagged Gln3 was significantly mislocalised in cells lacking the *ELP3* gene and accumulated in the nucleus (Figure 3C). Equally important and consistent with our phenotypic suppression data above (Figure 1D and Supplemental Figure 4) was our finding that Gln3 mislocalisation in the Elongator mutant could be efficiently suppressed by overexpressing tRNAs (tRNA^Gln^, tRNA^Lys^ and tRNA^Glu^) usually carrying U34 anticodon modifications (Figure 3C). This suggests it is a tRNA-related function and/or process that, when impaired or deficient due to loss of Elongator’s tRNA modification function, affects proper Gln3 localisation and subsequent transcriptional activation. Whether misregulated Gln3 localization accounts for the activated NCR response signature in the Elongator mutant is an attractive option since it entirely consists with a previous report from 2007 39 that placed Urm1 upstream of Gln3 and also showed Gln3 mislocalisation in an *urm1*Δ mutant, which by then was solely thought to be deficient in urmylation (a ubiquitin-like protein conjugation pathway) 39. However, with the recently advanced evidence showing that Urm1 has dual roles in protein urmylation and tRNA anticodon (U34) thiolation 8,9,10, Gln3 mislocalization shared between *elp3*Δ (Figure 3C) and *urm1*Δ 39 cells further supports our notion that it is loss of tRNA anticodon modification due to Elongator inactivation or U34 thiolation defects (rather than protein urmylation) which enhances NCR gene activation by Gln3, particularly under conditions of low TOR activity (Figure 3A and Supplemental Figure 5A).

**Figure 4:**
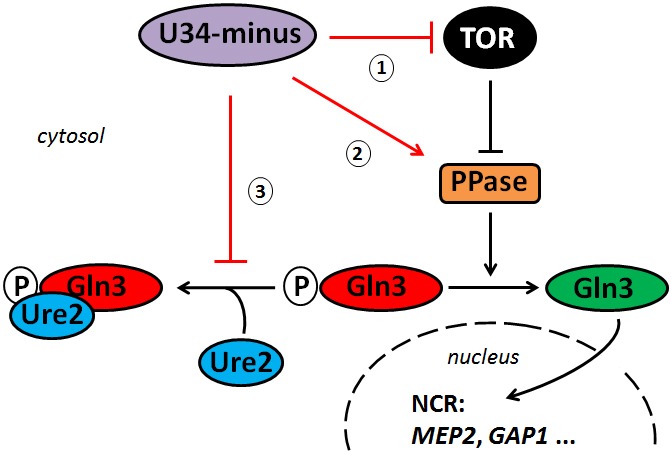
FIGURE 4: Working model for interactions between the TOR pathway and tRNA modifications. Loss of anticodon wobble uridine (U34) modifications enhances rapamycin sensitivity and NCR gene activation by Gln3 suggesting U34-minus mutants dampen TOR signaling. Such buffer function may operate upstream of TOR (1) or outside from TOR (2, 3) affecting steps that counter TOR-sensitive Gln3 inhibition. The latter (2, 3) may involve dephosporylation by a phosphatase (PPase) such as Sit4 (2) and/or Gln3 release from Ure2 (3) for mobilization and nuclear import. Alternatively, the effects of inappropriate U34 modifications on Gln3 misregulation could result from a combination of these options (1 - 3).

Our observation that improper tRNA modification deregulates transcription factor Gln3, is very reminiscent of two previous reports showing that Elongator-linked tRNA modification defects as well as KEOPS mutants with inappropriate N6-threonylcarbamoyl adenosine (t6A) modification in the anticodon stem loop can also misregulate transcription factor Gcn4 and the GAAC response associated with it 5,6. Intriguingly, this is in line with another study showing that a *sap185*∆*190*∆ mutant which lacks the Sit4 partner proteins, Sap185 and Sap190, caused *GCN4* induction, too 23. This is significant since *sap185*∆*190*∆ cells copy defects and phenotypes typical of *sit4*∆ and Elongator mutants including loss of tRNA modification and rapamycin hypersensitivity 23,27,28,29. Since protein dephosphorylation by Sit4 not only regulates Elongator’s tRNA modification function 27,28,29 but also operates on the two TOR pathway branches, NCR (Gln3) and GAAC (Gcn4) 15, it will be very important to study how the loss of tRNA modifications elicits Gln3 and Gcn4 response signatures at the molecular level. In yeast, almost all Elongator-linked phenotypes studied are rescued or at least partially suppressed by overexpressing tRNAs whose anticodons would normally carry the mcm5s2U34 modifications 10,40,41,42. Consistent with this, we found that overexpressing tRNA^Gln^, tRNA^Lys^ and tRNA^Glu^ is sufficient to suppress phenotypes (Figure 1D and Supplemental Figure 4) and properties that are typical of U34 modification mutants including Gln3 mislocalisation (Figure 3C). This strongly suggests that a translational defect in these tRNA modification mutants operates in deregulated TOR signaling. In support of this, a recent study has shown that U34 modifications indeed promote tRNA decoding functions during mRNA translation elongation 42.

Collectively, our data suggest that proper tRNA modifications play a positive role in the TOR signaling pathway. As a result of loss of anticodon modification, U34-minus cells undergo TOR suppression and enhance TOR-sensitive gene activation by Gln3 (NCR). Whether or not this operates upstream of TOR or outside the TOR network at a downstream level impinging on Gln3 activation, Ure2 interaction and/or nuclear import of Gln3 (Figure 4) is not entirely distinguishable from our preliminary evidence presented in this report. Alternatively, enhanced signaling in the U34 modification mutants may result from a combination of both options (Figure 4). In support of such scenario, our data show that drastic NCR gene activation by Gln3 in *elp3*Δ cells requires TOR inhibition by rapamycin for Gln3 mobilisation in concert with the Sit4 phosphatase, which (in addition to release of Gln3 from Ure2) further contributes to nuclear import of Gln3 and NCR gene transcription activation by Gln3. Whether the enhanced Gln3 activation (together with Gcn4 induction reported previously 5,6,43) in tRNA modification mutants reflect cellular (stress) responses to overcome scenarios typically encountered upon TOR inhibition or suppression, i.e. poor nitrogen supply (NCR) or amino acid depletion (GAAC), are attractive hypotheses that need to be addressed in further studies. Given a report, however, that amino-acyl-tRNA synthetases have been found upregulated in tRNA modification mutants 5, tRNA charging defects, which may be associated with loss of U34 modifications, could potentially feed into TOR pathway signaling.

## MATERIALS AND METHODS

### General methods, yeast growth and strains

Routine yeast growth was in yeast extract, peptone, and dextrose (YPD) rich or synthetic complete (SC) minimal media 44. For TOR modulation, growth media were supplemented with proline (poor N-source) or glutamine (good N-source). For testing the effect of TOR inhibitor drugs, 2.5 - 150 nM rapamycin (Calbiochem) or 2.5 - 15 mM, caffeine (Sigma) were added to YPD medium, and yeast growth was monitored for 3 - 5 days at 30°C. Yeast transformation used the PEG-lithium-acetate method 45 and tRNA overexpression involved multicopy plasmids pKQ/E carrying tRNA^Lys^UUU, tRNA^Gln^UUG and tRNA^Glu^UUC genes 10. To assess the response to lethal induction of zymocin’s tRNase subunit (γ-toxin), strains were transformed with pHMS14 12, and growth was followed under conditions of tRNase expression (galactose) and repression (glucose). Substitutions of Sit4 proline residue 187 to either alanine or phenylalanine were generated using the two-step fusion PCR approach 46. Mutants generated in this study by one-step PCR mediated gene deletion 47 derived from previously described strain backgrounds and used knock-out primers specific for *ELP3*, *KTI12*, *URM1*, *NCS2* and *GLN3 *12,13,17.

### RT-PCR and qPCR methods

Total yeast RNA isolation and RT-PCR were carried out as previously described 36 with the following oligonucleotides *ACT1*-FW (5’-CTT CCG GTA GAA CTA CTG GT-3’); *ACT1*-RV (5’-CCT TAC GGA CAT CGA CAT CA-3’); *GAP1*-FW (5’-TCC CGC TTC GCT ACT GAT TG-3’); *GAP1*-RV (5’-GCA GAG TTA CCG ACA GAT AA-3’); *MEP2*-FW (5’-GGT ATG TTT GCC GCA GTC AC-3’) and *MEP2*-RV (5’-ACC ACC CAC ACC ATG GAT AG-3’). Real-time PCR used a Mastercycler (Eppendorf), the SensiFAST™ SYBR® No-ROX Kit (BIOLINE) and the *ALG9* standardization protocol 38. qPCR involved *ALG9*-qFW (5’-GTC ACG GAT AGT GGC TTT GG-3’); *ALG9*-qRV (5’-TGG CAG CAG GAA AGA ACT TG-3’); *MEP2*-qFW (5’-GTA TGT TTG CCG CAG TCA CC-3’) and *MEP2*-qRV (5’-CAG ACC CAG CAT GCA ATA GG-3’) oligonucleotides.

### Cellular localization of Gln3-GFP

Yeast strains carrying pRS416-GFP-Gln3 48,49 were grown in yeast nitrogen base media with glutamine as the sole N-source, left untreated or treated with 10 nM rapamycin for 20 min. Subsequently, cells were fixed by adding 3.7% formaldehyde directly to the medium and incubated for 10 min at room temperature and washed once with water. Cells were resuspended in water containing 1 µg•ml^-1^ 4,6 diamidino-2-phenylindole (DAPI, Sigma, Germany). Following washing with water, cells were analyzed using an Olympus BX53 microscope with appropriate filters for DAPI and GFP fluorescence. Images were captured using the CellSens 1.6 software package (Olympus).

## SUPPLEMENTAL MATERIAL

Click here for supplemental data file.

All supplemental data for this article are also available online at http://microbialcell.com/researcharticles/loss-of-wobble-uridine-modification-in-trna-anticodons-interferes-with-tor-pathway-signaling/.
