# Supplementary material for: Loss of wobble uridine modification in tRNA anticodons interferes with TOR pathway signaling
Source: Microb Cell. 2014 Nov 29;1(12):416–24. doi: 10.15698/mic2014.12.179 (PMC5349137; doi:10.15698/mic2014.12.179)
Supplement: Supplementary file 1 [file mic-01-416-s01.pdf]

Supplemental Data

Microbial Cell Research Report (MIC-0174E178)

Loss of wobble uridine modification in tRNA anticodons interferes with TOR pathway signaling

Viktor Scheidt[1§], André Jüdes[1§], Christian Bär[1,2§], Roland Klassen[1] and Raffael Schaffrath[1]*

[1] Institut für Biologie, Abteilung Mikrobiologie, Universität Kassel,
D-34132 Kassel, Germany

[2] Present address: Molecular Oncology Program, Spanish National Cancer Centre (CNIO), Melchor Fernandez Almagro 3, Madrid, Spain

§ These authors contributed equally to the study

* Corresponding author:

Raffael Schaffrath
Institut für Biologie, Abteilung Mikrobiologie
Universität Kassel, D-34132 Kassel, Germany

Phone: +49-561-804-4175
FAX: +49-561-804-4337
eMail: schaffrath@uni-kassel.de

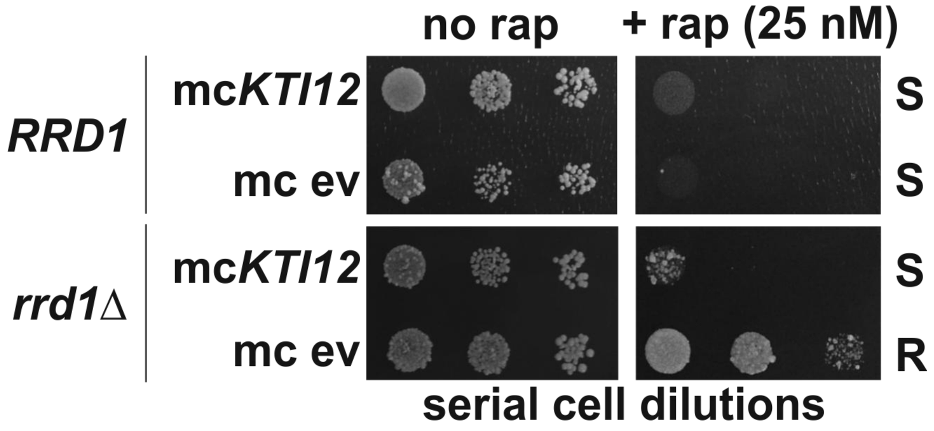

Figure S1. Resistance towards growth inhibition by rapamycin of the TOR signalling mutant *rrd1*Δ requires normal *KTI12* gene copy number and is suppressed multi-copy *KTI12*. As indicated, ten-fold serial dilutions of *RRD1* and *rrd1*Δ yeast strains carrying multi-copy empty vector (mc ev) controls or the *KTI12* gene on a multi-copy (mc*KTI12*) plasmid were spotted onto YPD media supplemented with rapamycin (25 nM) or no TOR inhibitor drug (no rap). Cultivation was for 3 days at 30 °C. Growth in the presence of rapamycin equals drug resistance (R), lack of (or significantly suppressed) growth indicates sensitivity (S).

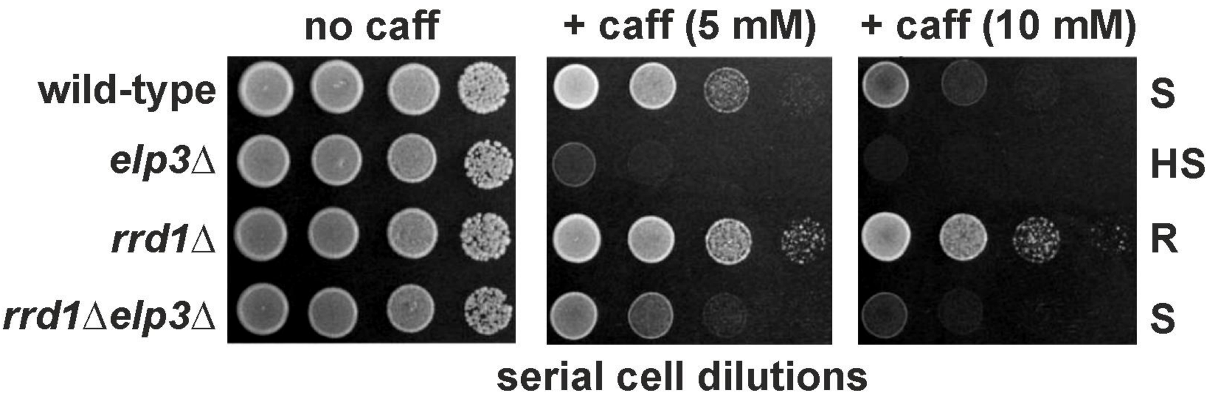

Figure S2. Loss of tRNA modification in an Elongator mutant (*elp3*Δ) confers caffeine hyper-sensitivity, a trait epistatic over an *rrd1*Δ null-allele, which alone confers caffeine resistance. Ten-fold serial dilutions of the indicated yeast tester strains were spotted onto YPD media supplemented without caffeine (no caff) and various doses (5 and 10 mM) of the TOR inhibitor drug (+ caff). Following growth for 3 days at 30 °C, drug resistant (R) responses were distinguishable from sensitive (S) and hyper-sensitive (HS) ones.

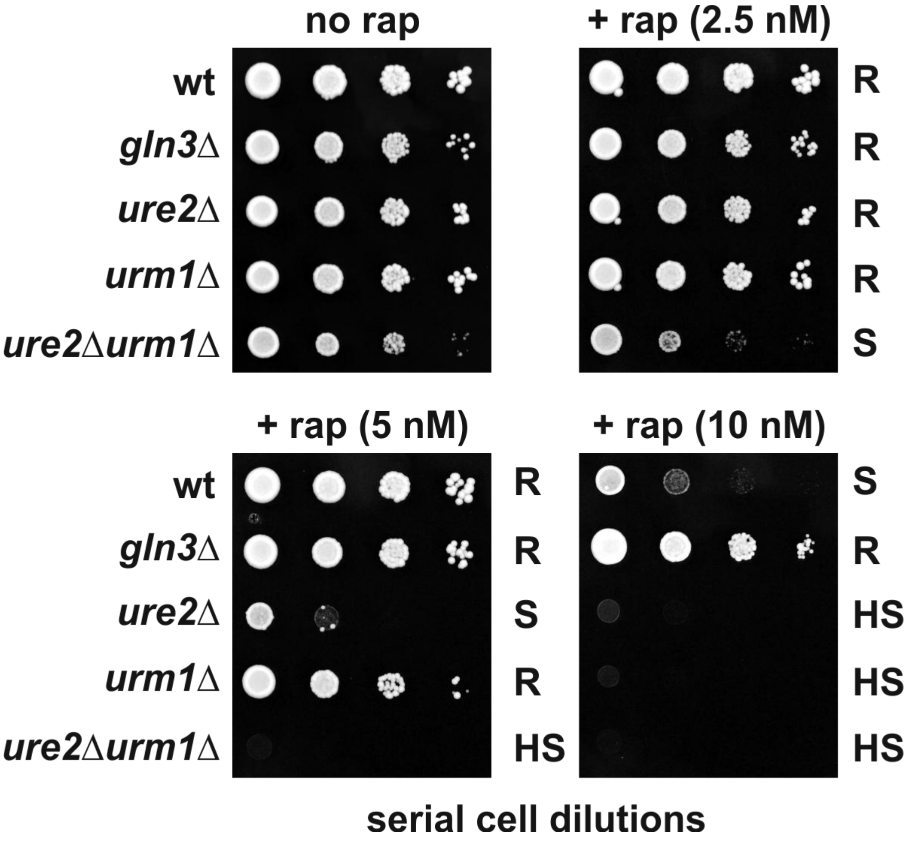

Figure S3. In tandem with an *urm1*Δ-linked U34 anticodon thiolation defect, the rapamycin hyper-sensitivity due to loss of *URE2* gene function becomes aggravated. Ten-fold serial dilutions of the indicated yeast tester strains were spotted onto YPD media containing no rapamycin (no rap) or various doses (2.5, 5 and 10 nM) of the TOR inhibitor drug (+ rap). Yeast cultivation was for 3 days at 30 °C. Growth in the presence of the drug indicates rapamycin tolerance or resistance (R), while lack of growth equals sensitivity (S) or hyper-sensitivity (HS) towards the drug.

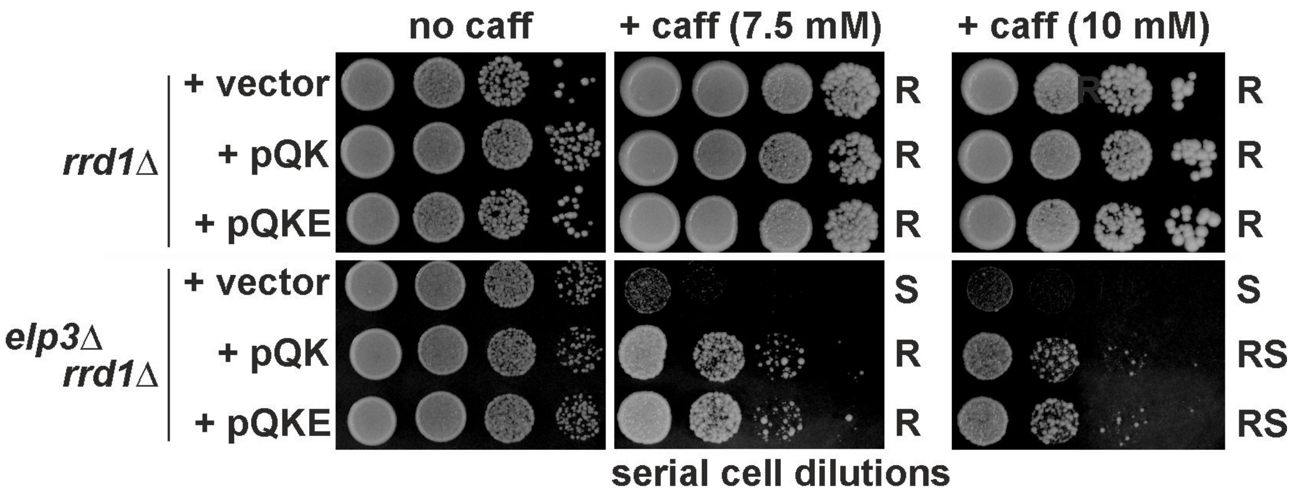

Figure S4. Overexpression of Elongator-dependent tRNA species (tRNA^{Gln} [Q], tRNA^{Lys} [K] and tRNA^{Glu} [E]) suppresses caffeine sensitivity, a phenotype typical of *elp3*Δ single and *elp3*Δ*rrd1*Δ double mutants (see also Figure S2). Ten-fold serial dilutions of the indicated yeast strains carrying empty vector controls (vector) or plasmids with genes for tRNA^{Gln}, tRNA^{Lys} and/or tRNA^{Glu} (pQKE/pQK) were spotted onto YPD media without (no caff) and with caffeine (+ caff 7.5 and 10 mM). Following growth for 3 days at 30 °C, drug resistance (R) and sensitivity (S) are distinguishable from reduced sensitive (RS) responses that result from phenotypic suppression by the tRNA overexpression plasmids, pQKE and pKQ.

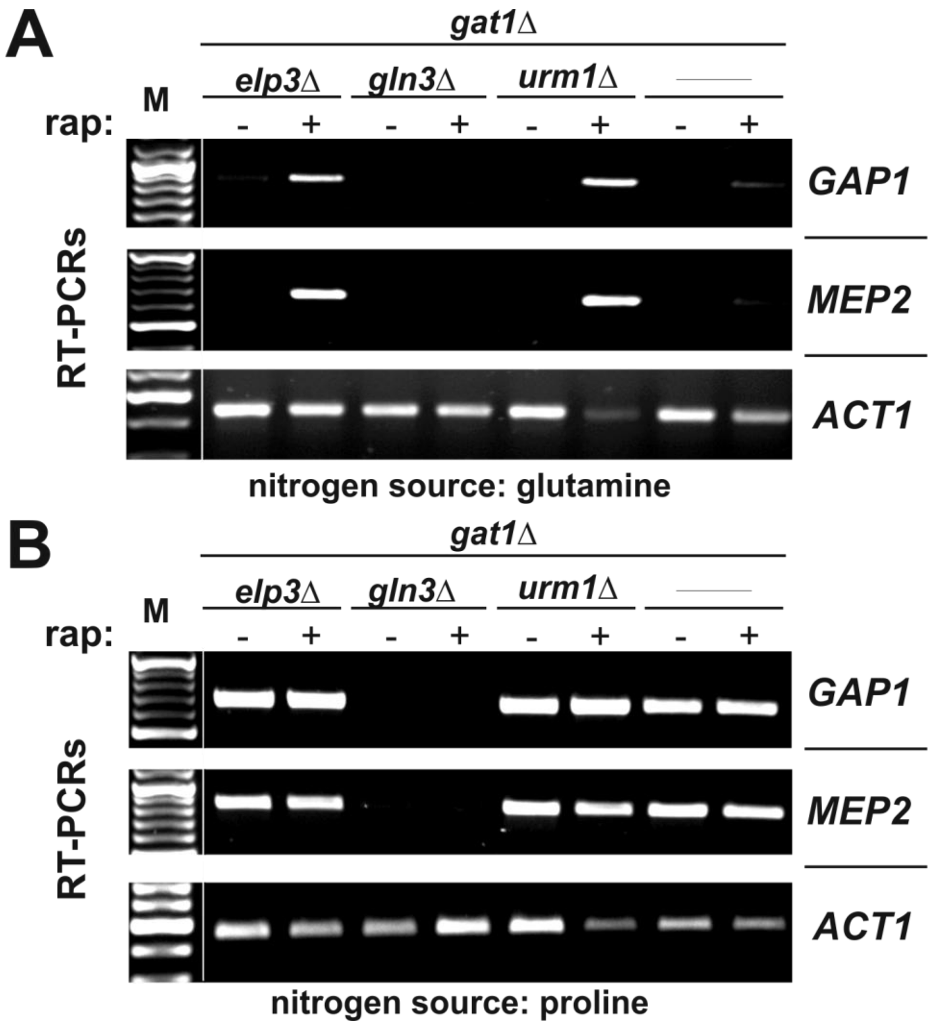

Figure S5. RT-PCRs reveal *MEP2* and *GAP1* gene transcription by Gln3 is enhanced in anticodon modification mutants that lack Elongator (*elp3*Δ) or U34 thiolation (*urm1*Δ) activities. Total RNA was isolated from the indicated strains cultivated in the absence (-) or presence (+) of 50 mM rapamycin (rap) with good (glutamine) (A) or poor (proline) nitrogen sources. Following RT-PCR, *MEP2* and *GAP1* gene transcription by Gln3 was analysed in comparison to actin (*ACT1*) gene expression. Note that *MEP2* and *GAP1* transcription induction by Gln3 particularly occurs under good nitrogen supply (A: glutamine) and in response to TOR inhibition by rapamycin, while under conditions of TOR suppression by a poor nitrogen source (B: proline) NCR gene transcription is constitutive and slightly increased in relation to basal wild-type levels.
